# Ecological, Functional, and Phylogenetic Determinants of Cyanobacterial Biomineralisation

**DOI:** 10.1111/1758-2229.70281

**Published:** 2026-01-27

**Authors:** Federica Tiddia, Sandeesha Kodru, Dario Piano, Domenica Farci

**Affiliations:** ^1^ Department of Botany and Plant Physiology Warsaw University of Life Sciences—SGGW Warsaw Poland; ^2^ Plant Physiology and Photobiology Group, Department of Life and Environmental Sciences University of Cagliari Cagliari Italy

**Keywords:** biomineralisation, CaCO_3_, carbon cycle, carbon fixation, carbonate precipitation, cell envelope, microbial carbon sequestration

## Abstract

Cyanobacteria play a key role in the biomineralisation of carbon dioxide into solid carbonates, a critical process in the global carbon biogeochemical cycle that links atmospheric CO_2_ to lithospheric carbonate reservoirs. While photosynthetic carbon fixation by these microorganisms has been extensively studied and is relatively well understood, the biomineralisation pathway remains much less explored, likely leading to an underestimation of its global relevance. This review summarises current findings and highlights the ecological and cellular factors that contribute to cyanobacterial biomineralisation. In particular, the need to cope with fluctuating environmental conditions has played a central role in enabling cyanobacteria to develop rapid metabolic adaptations together with the evolution of a complex cell wall architecture. Within this framework, biomineralisation emerged as a tangible and effective adaptive strategy. Particular attention is given to the metabolic processes and related ion trafficking mechanisms across the cell envelope, which are instrumental in facilitating mineral nucleation and growth.

## Graphical abstract

Cyanobacteria contribute to the global carbon cycle by biomineralising CO_2_ into solid carbonates. Unlike photosynthesis, biomineralisation is a less‐understood, multifaceted and adaptive metabolic process with significant global impact. It involves cell wall structures, genetic factors, and metabolic pathways that enable mineral formation and help cyanobacteria respond to environmental changes, highlighting the ecological importance of this process.

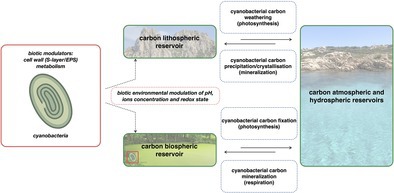

## 1 Introduction

Biomineralisation is the biological process through which organisms generate and deposit minerals, either amorphous or crystalline, via mechanisms that may be either biologically controlled or biologically induced but environmentally driven. This phenomenon is widespread across the tree of life, occurring in prokaryotes and eukaryotes. Bacteria and cyanobacteria in particular have been among the most influential agents shaping mineral formation on Earth, both throughout geological time and across diverse environments. This phylum of oxygenic phototrophic prokaryotes has significantly changed Earth's biosphere over nearly four billion years. Ubiquitous in aquatic ecosystems, they contribute to biogeochemical cycling of macronutrients, micronutrients, water and to aquatic photosynthesis, which accounts for approximately 50%–80% of global carbon fixation. As a reference, *Prochlorococcus*, a single genus, accounts for nearly 20% of global photosynthetic carbon capture (Jansson and Northen 2010). Beyond aquatic systems, many cyanobacteria colonise extreme terrestrial environments, including deserts, polar regions and thermal springs, where they influence local biogeochemical cycles and ecosystems (Chrismas et al. 2015; Whitton and Potts 2012). Cyanobacteria have been instrumental in transforming Earth's early atmosphere, with their evolution marking the transition from a reducing to an oxidising atmosphere during the great oxygenation event (GOE), enabling the formation of oxidised minerals and expanding the range of chemical interactions between the biosphere and the geosphere. This global shift not only allowed aerobic metabolism but also played a key role in shaping mineral weathering, soil formation and nutrient cycling processes (Gross 2015; Hazen et al. 2008; Svirčev et al. 2019).

**FIGURE 1:**
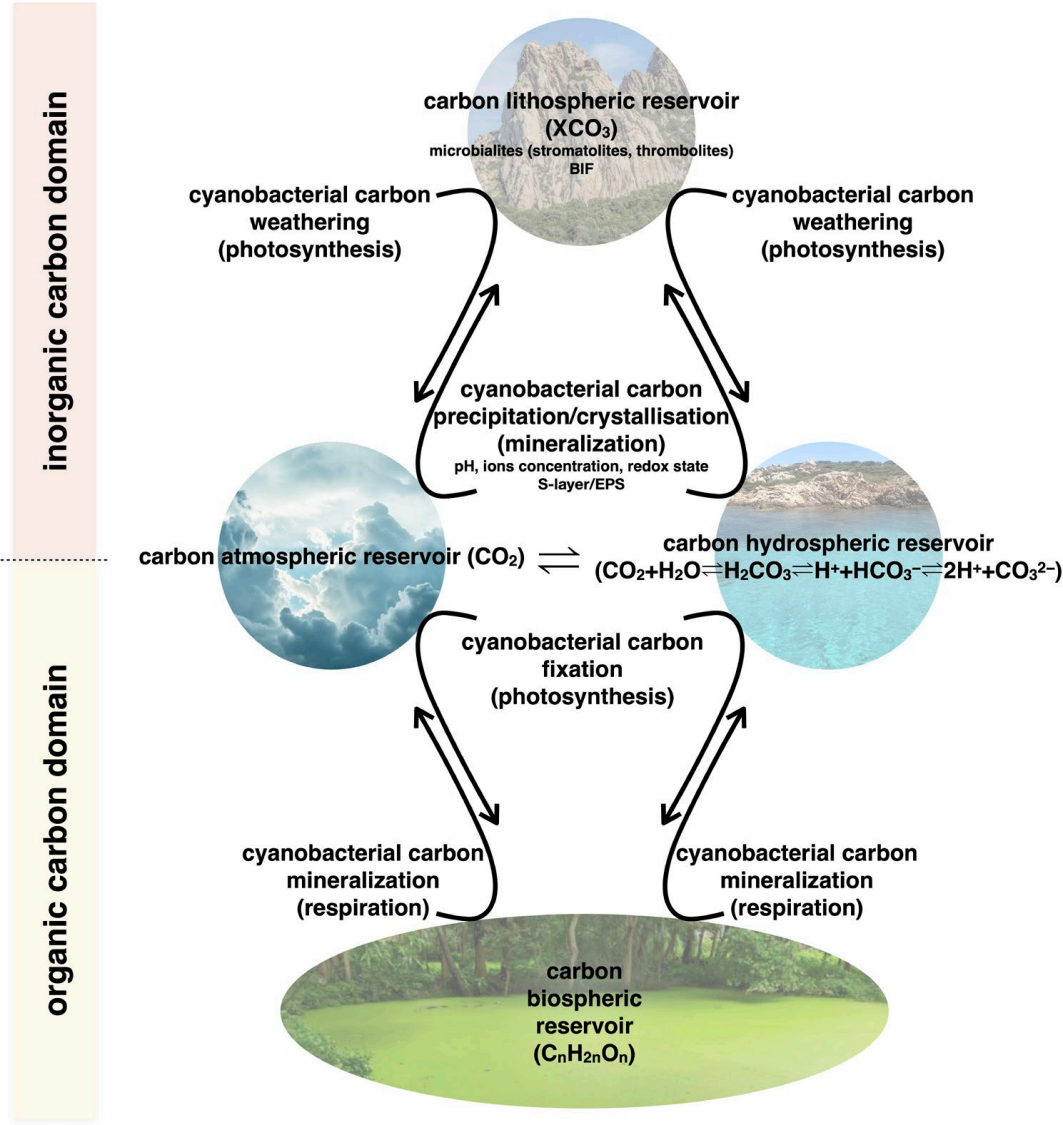
Cyanobacterial metabolism and global carbon reservoirs. Atmospheric and hydrospheric carbon reservoirs are in constant exchange and indirectly linked to lithospheric and biospheric ones. Cyanobacteria influence these carbon flows through key metabolic processes such as photosynthetic carbon fixation and biologically mediated carbon mineralisation. By altering carbonate chemistry in ways that *promote carbonate precipitation*, they facilitate the transfer of inorganic carbon from the atmosphere into the lithosphere and hydrosphere as solid mineral phases (weathering). Conversely, they can induce carbonate precipitation, crystalline or amorphous, driven by pH shifts, ion concentrations, redox conditions and cell surface features such as the S‐layer and EPS. Through photosynthesis, cyanobacteria fix inorganic carbon from the atmosphere and hydrosphere into organic matter (carbon fixation). Through respiration, they mineralise organic carbon, returning it to inorganic form and releasing it back into the atmospheric and hydrospheric reservoirs.

Within the carbon biogeochemical cycle, cyanobacteria are important for transferring carbon from its atmospheric form as CO_2_ to the lithosphere and hydrosphere through two uniquely coexisting processes: photosynthetic carbon fixation and biologically mediated carbon mineralisation. These activities alter carbonate chemistry, drive CO_2_ sequestration and promote saturation conditions that lead to carbonate precipitation and long‐term storage in sedimentary rocks over geological timescales, a process that influenced global climate regulation for billions of years and continues today (Figure 1) (Benzerara et al. 2010). For instance, stromatolites, sedimentary manganese‐enriched carbonate structures of fossil origin, resemble modern products of cyanobacterial activity. These formations are key palaeobiological archives, preserving information about early microbial life in its environment, some of the earliest known examples of organisms shaping their surroundings to buffer extreme environmental conditions (Olejarz et al. 2021; Tice and Lowe 2004).

Cyanobacterial biomineralisation primarily involves the production of calcium carbonate (CaCO_3_), but also affects other mineral systems by modifying local chemistry through shifts in pH, ion availability, or redox state.

Although often associated with eukaryotic organisms such as corals and molluscs, biomineralisation is a widespread microbial phenomenon with a deep evolutionary history and ongoing ecological relevance (Kamennaya et al. 2012). While it may have initially emerged as a metabolic byproduct, it now represents an active adaptive strategy enabling microbes to alter their microenvironment, stabilising extracellular conditions and promoting survival. In cyanobacteria, biomineralisation reflects a complex interplay between their physiology and the environment. This process manifests in various forms, broadly classified by the nature of the minerals produced and their spatial association with the cells. In this group of microorganisms, mineral precipitation can confer several ecological and physiological advantages: it can buffer external pH, regulate the availability of inorganic carbon and help stabilise local microenvironments, thereby supporting core metabolic processes and enhancing habitability in fluctuating or extreme conditions. Interactions with geological minerals can provide structural support or serve as sources of elements and substrates for redox reactions, potentially ‘reshaping’ the mineral and playing important roles in early cyanobacterial evolution. Abiotic chemicals and physical factors, including ion availability, pH and redox state, influence and are modulated by the cell envelope, a functional interface between the cell and the environment promoting mineral nucleation. Together, these biotic and abiotic factors define an articulated framework that remarks the diversity and complexity of cyanobacterial biomineralisation.

Despite the progress in characterising cyanobacterial taxa, cellular localisation and environments associated with biomineralisation, their physiological and mechanistic foundations remain largely unclear. Key open questions concern the molecular pathways controlling bio‐minerals nucleation and growth, the evolutionary pressures that preserved these traits, and their ecological roles beyond passive CaCO_3_ deposition. Emerging evidence suggests potential links to stress tolerance, nutrient storage and biofilm formation (Benzerara et al. 2014; Popall et al. 2020). This limited understanding highlights the complexity of biomineralisation, its potentially broad influence on cyanobacteria physiology and the complex environmental interactions involved (Table 1).

**TABLE 1:** Examples of biomineral types and associated cyanobacterial functions. The table shows, for each different biomineral, the role of cyanobacteria in the process, the associated function and the environments in which it occurs.

Biomineral	Cyanobacterial role	Function/Significance	Environmental setting	References
Calcium carbonate (CaCO_3_)	Cell surface‐mediated nucleation	Carbon sink, pH buffering	Buffering marine mats, stromatolites	Dupraz and Visscher (2005); Dupraz et al. (2009); Popall et al. (2020); Landing and Johnson (2024).
Iron oxides	Cell surface‐catalysed redox reactions	Electron transfer, detoxication	Hydrothermal vents, soils	Jiang et al. (2013); Huang et al. (2022)
Silicates	Passive incorporation	UV shielding, stress resistance	Desert crusts	Popall et al. (2020); Jehlička et al. (2024).
Manganese minerals	Possibly active precipitation	Ancient bio‐mats, oxidative stress	Fossil record, stromatolites	Tice and Lowe (2004).

This review summarises the current understanding of cyanobacterial biomineralisation by (i) outlining phylogenetic and metabolic traits on its occurrence; (ii) examining the spatial localisation and functional roles of biominerals in relation to the cell; (iii) tracing its evolutionary origins from early Earth to modern global cycles and (iv) evaluating how diverse environmental factors, cell envelope structures and ion homeostasis shape biomineralisation across taxa. We conclude by considering how these processes contribute to the ecological resilience of cyanobacteria and the environments they inhabit.

## 2 Phylogenetic and Metabolic Traits of Cyanobacterial Biomineralisation

Cyanobacteria are direct descendants of the ancient photosynthetic organisms that initiated oxygenic photosynthesis, profoundly influencing the Earth's atmosphere. Beyond photosynthesis, they also play a crucial role in biomineralisation, particularly in the precipitation of CaCO_3_ in both aquatic and soil environments. Stromatolites are paleontological evidence of this activity, a primary reference for early chronobiology in the geological time scale, indicating the early emergence of biomineralisation during cyanobacterial evolution alongside the rise of oxygenic photosynthesis. At present, unicellular and colonial cyanobacteria, such as *Gloeocapsa* and *Pleurocapsa*, exhibit significant calcifying potential and contribute to the formation of microbialites, sedimentary structures produced by microbial activity, particularly through CaCO_3_ precipitation. These include stromatolite‐like formations and thrombolites, which, even if less represented with respect to the past, are still produced nowadays as aragonite via the extracellular carbonate deposition (Couradeau et al. 2011; Della 2015; Gérard et al. 2013). Similar formations seem to be strictly dependent on the cell structures facing the environment, such as the extracellular polymeric substances (EPS) on the cell envelope. Extremophile cyanobacteria in hot springs secrete thick EPS layers that promote extracellular carbonate nucleation under high temperatures (Kanellopoulos et al. 2022). In freshwater ecosystems, filamentous heterocyst‐forming species like *Calothrix* sp. and *Nostoc commune* also induce CaCO_3_ precipitation through EPS matrix (Benzerara et al. 2021; de Brito et al. 2023). Historically, such ecological adaptations likely supported global stromatolites formation influencing Earth's biogeochemical cycles and the carbon lithospheric reservoir (Figure 1).

But how early did this process emerge in the evolutionary history of the phylum? And in which cyanobacterial groups is biomineralisation most prevalent? Phylogenetic studies based on 16S rRNA and whole‐genome sequencing show that biomineralising cyanobacteria are mainly found in early‐branching and benthic clades (Ludwig and Schleifer 1994; Magnabosco et al. 2024; Mehdizadeh Allaf and Peerhossaini 2022; Ragon et al. 2014; Woese 1987). Groups of ancient origin, such as *Gloeobacter* and *Pleurocapsa*, often associate with sediment surfaces and produce thick EPS layers that promote mineral precipitation. Their basal position in the cyanobacterial tree suggests biomineralisation as an ancient trait, likely present early in cyanobacterial evolution, thereby linking it to the global formation of stromatolites.

**FIGURE 2:**
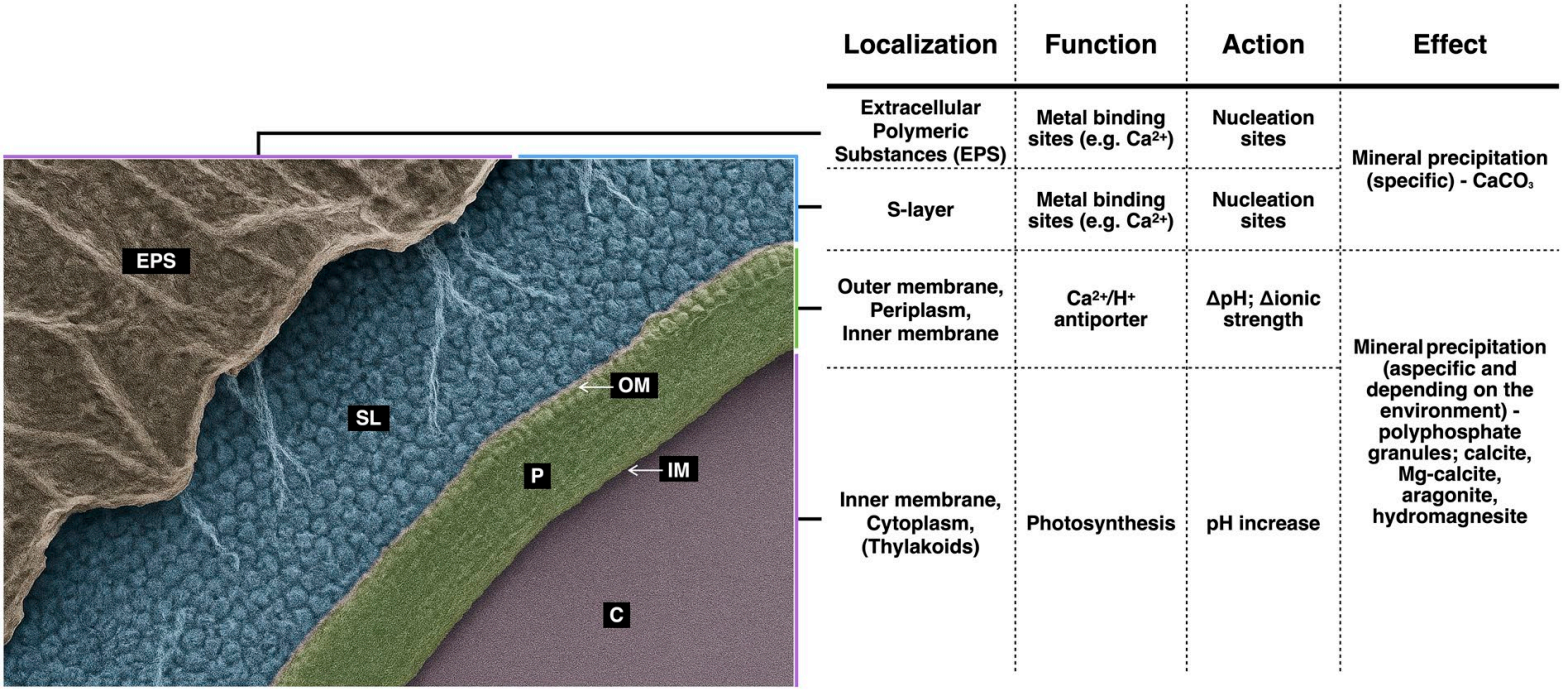
Representative cyanobacterial cell wall organisation. The image shows the sequence of structural layers isolating the cytosol (C) from the external environment: the inner membrane (IM), the periplasmic space (P), the outer membrane (OM), the S‐layer (SL) and the extracellular polymeric substances (EPS). The S‐layer and EPS form the outermost boundary and play a pivotal role in biomineralisation.

Initially thought to occur rarely and only extracellularly, biomineralisation is widespread and also occurs intracellularly (Benzerara et al. 2014; De Wever et al. 2019; Li et al. 2016; Segovia‐Campos et al. 2022). Ecological and metabolic factors, alongside chemical and physical conditions, drive biomineralisation. Key abiotic factors include ion availability and concentration, particularly calcium, dissolved inorganic carbon (DIC), pH, redox potential, saturation levels and anion presence. Beyond these abiotic factors, cyanobacteria accelerate mineral formation by altering the local environmental pH and carbonate chemistry through photosynthesis and ion exchange (Jiang et al. 2013). Cell structures, particularly the S‐layer and the EPS matrix, provide nucleation sites by their organised charged surfaces capable of binding metal ions and other mineral precursors, effectively concentrating and orienting them to promote nucleation (Obst et al. 2009; Paulo et al. 2018). In *Synechocystis*, the S‐layer facilitates nucleation through its paracrystalline isoporous structure and associated charge patterns, enhancing Ca^**2**+^ attraction (Karlsson et al. 1983; Liang et al. 2013; Schultze‐Lam et al. 1992; Schultze‐Lam and Beveridge 1994). This is consistent with findings that cell surface components can mediate cell‐mineral interactions and regulate the specificity and distribution of crystal formation (Gilbert et al. 2005). The EPS matrix, rich in polysaccharides, proteins and other biopolymers, traps ions, altering pH and stabilising nascent mineral phases, thereby accelerating crystallisation and influencing mineral morphology. Together, S‐layer and EPS create micro‐environments that favour the initiation of bio‐minerals, influence their structural properties and regulate their growth (Figure 2).

The model cyanobacterium *Synechocystis* sp. PCC 6803 influences CaCO_3_ precipitation, mineral formation, morphology and distribution under controlled environmental parameters, such as calcium and magnesium concentrations (Han et al. 2017). Through surface structures and envelope‐associated proteins such as Ca^2+^/H^+^ antiporters, these organisms modify their extracellular environment, also promoting supersaturation (Jiang et al. 2013) (Figure 2). Supersaturation, arising when ion concentrations exceed solubility, is central to both extracellular and intracellular biomineralisation. Photosynthesis and metabolic activity drive local supersaturation by varying local environmental pH and ion availability, triggering crystal nucleation and growth. For instance, in phosphate‐rich extracellular solutions, polyphosphate granules can concentrate Ca^2+^, and their subsequent hydrolysis may locally increase the ion activity to the point of CaCO_3_ supersaturation. CaCO_3_ inclusions can then form within or around these granules, which also function as phosphate storage, linking mineral formation to metabolic activity (Li et al. 2016). Via supersaturation, the precipitation of calcite, Mg‐calcite, aragonite, and hydromagnesite is also controlled, depending on the chemical environment (McCutcheon et al. 2014; Spitzer et al. 2015). Similarly, the activity of microbial communities may contribute to carbonate precipitation both in the environment and directly on the surface of cyanobacteria cells (Couradeau et al. 2013) (Figure 2).

## 3 Biominerals' Localisation and Functional Relationship With the Cyanobacterial Cell

Biominerals in cyanobacteria have considerable diversity, making it difficult to classify all cases within a single framework. This diversity reflects a complex interplay between species‐specific metabolic traits and the environmental conditions. Because environmental factors can exert a strong and sometimes dominant influence on mineral formation, distinguishing the true origin of a given biomineral, whether primarily metabolic, environmental, or a combination of both, becomes particularly challenging.

**FIGURE 3:**
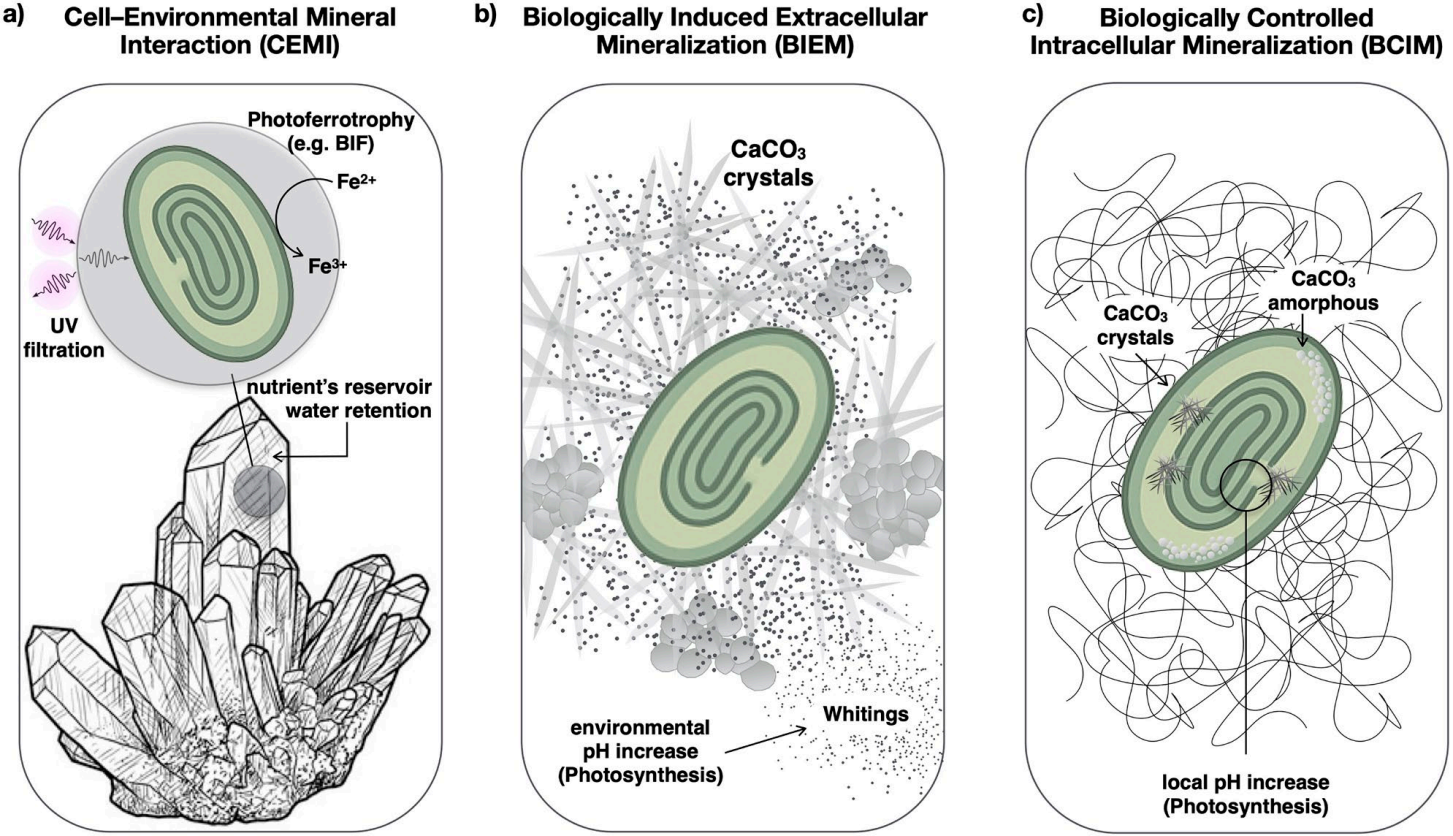
Schematic of the three main types of interactions between crystals and cyanobacterial cells. The panel (a) illustrates how biogenic crystals of nonbiotic origin can be passively exploited by cyanobacteria, as UV shields, electron donors (e.g., in photoferrotrophy), or simple structural supports. The panel (b) shows extracellular mineralisation, where metabolic activity, such as photosynthesis, raises the local pH around the cell, inducing carbonate precipitation (e.g., whitings). The panel (c) depicts intracellular biomineralisation, where certain cyanobacteria actively control the precipitation of amorphous or crystalline carbonates. In this case, the pH modulation by photosynthetic activity also plays a key role in driving internal precipitation.

Nonetheless, several reference points can be identified to provide a rational overview of this heterogeneous scenario. For example, CaCO_3_ represents the most commonly observed mineral phase (Couradeau et al. 2013; De Wever et al. 2019; Görgen et al. 2021; Segovia‐Campos et al. 2022) and, more broadly, biomineralisation processes can be classified based on the chemical nature of the minerals produced and their spatial association with the cell (Figures 2 and 3).

### 3.1 Cell–Environmental Mineral Interaction (CEMI): Topology, Diversity, and Functions

Cyanobacteria do not merely coexist with minerals but interact with them through a structural and metabolic continuum. Depending on the CEMI'S features, it is possible to understand ecological strategies, trace evolutionary patterns and identify mineral influences on survival and metabolism. Some interactions represent passive mineral associations, such as endolithic colonisation or surface adsorption, where cyanobacteria exploit pre‐existing geological substrates without directly controlling mineral formation. These minerals may provide physical support and UV protection, or have metabolic roles, as reservoirs of essential elements (e.g., Fe, Mn, Ca), water and substrates for redox reactions supporting microbial energy conservation (Tables 2 and 3). Clear structural examples are endolithic communities in deserts. *Chroococcidiopsis* spp. colonise translucent minerals like quartz and gypsum, which filter harmful UV radiation while allowing visible light for photosynthesis and trapping microfilms of water, providing protection in hyper‐arid environments (Jehlička et al. 2024) (Figures 3a, Tables 2 and 3).

**TABLE 2:** Comparison between CEMI, BIEM, and BCIM in cyanobacteria. The table compares biomineralisation types, highlighting the localisation, regulation, function, species, and crystal type.

Feature	CEMI	BIEM	BCIM
Mineral localisation	Extracellular (pre‐existing)	Extracellular	Intracellular
Regulation	Genetic/metabolic control	Indirect/metabolic byproduct	Genetic/metabolic control
Functions	UV shielding, source of water, reducing power and nutrients	Detoxification, UV protection, pH regulation	Ion storage, pH buffering, metabolic regulation
Reported species	*Chroococcidiopsis* sp., *Synechococcus* spp., *Osciallatoria spp*	*Microcoleus chthonoplastes* , *Nostoc spp*, *Synechococcus* spp., *Gloeocapsa* sp.	*Thermosynechococcus elongatus, Gloeomargarita lithophora*, *Synechocystis* spp. (e.g., *S. calcipolaris*)
Crystal type	Quartz, gypsum, ferrihydrite and haematite	Mostly calcite/aragonite	Amorphous or crystalline CaCO_3_, also with Sr., Mg, Ba inclusions
References	Vaara (1982); Liang et al. (2013); de Brito et al. (2023); Jehlička et al. (2024).	Konhauser (2007); Dupraz et al. (2009); Zhang et al. (2019); Bundeleva et al. (2014); de Brito et al. (2023).	Karlsson et al. (1983); Jiang et al. (2013); Benzerara et al. (2014); Görgen et al. (2021).

**TABLE 3:** Most peculiar and reported mineral types and their localisation in cyanobacterial species. The table shows, for each cyanobacterial species, mineral, localisation and environment in which they have been shown to perform biomineralisation.

Species	Mineral	Localisation	Environment	References
*Chroococcidiopsis* sp.	Quartz, gypsum, and arenites	CEMI (Endolithic)	Desert	Vaara (1982); Jehlička et al. (2024)
*Synechococcus* sp.	CaCO_3_	BIEM (Extracellular)	Aquatic	Karlsson et al. (1983); Lee et al. (2006); Obst et al. (2009); Liang et al. (2013)
*Gloeomargarita lithophora*	Intracellular carbonate	BCIM (Intracellular, random)	Freshwater	Benzerara et al. (2014); Moreira et al. (2017).
*Synechococcus calcipolaris*	CaCO_3_	BCIM (Intracellular, polar)	Marine	Benzerara et al. (2014)
*Microcystis aeruginosa PCC 7806*	Amorphous CaCO_3_	BCIM (Intracellular)	Freshwater	Bruley et al. (2025)

Metabolically, *Synechococcus* spp. and *Oscillatoria* spp. are known to interact with Fe‐rich minerals, such as ferrihydrite and haematite, under anoxic conditions. Several studies hypothesised that ancestral cyanobacterial may have similar interactions for using Fe^**2**+^ as an electron donor in anoxygenic photosynthesis, a process known as photoferrotrophy. This ancestral pathway could have contributed to precambrian banded iron formations (BIF) (Camacho et al. 2017; Swanner et al. 2015) (Figure 3a, Tables 2 and 3). Such mineral‐microbe interactions likely supported energy conservation in early low‐oxygen environments and influenced iron and sulphur cycles. Moreover, they may have enhanced metabolic versatility in ancestral cyanobacteria, promoting colonisation of mineral‐rich niches and adaptive radiation (Camacho et al. 2017).

In conclusion, two main coexisting factors explain the persistence of CEMI: minerals provide a structural/protective/physical support and are sources of essential elements with trophic roles as substrates for redox reactions (Figure 3a). The mineral–microbe interface thus represents an ancestral space critical for metabolic divergence, particularly before oxygenic photosynthesis, when alternative electron donors like Fe^**2**+^ and reduced sulphur compounds were central to energy metabolism.

### 3.2 Biologically Induced Extracellular Mineralisation (BIEM): Topology, Diversity, and Functions

In BIEM, the organism's metabolism alters the local micro‐environment triggering mineral nucleation, typically on the cell surface (Tables 2 and 3). This represents an active, biologically controlled form of mineralisation, where metabolic processes such as photosynthesis, respiration, ion sequestration, or pH regulation directly promote extracellular mineral formation.

BIEM is widespread in cyanobacteria, including *Chroococcidiopsis, Pleurocapsales, Microcoleus chthonoplastes*, *Nostoc* spp. and *Synechococcus* spp. Metabolic activities, particularly photosynthesis and respiration, increase local environmental pH and modify ion concentrations, promoting extracellular precipitation of carbonates in various mineral forms, including calcite, aragonite and other carbonate phases and/or iron oxides (Dupraz et al. 2009; Konhauser 2007; Zhang et al. 2019) (Tables 3 and 4).

**TABLE 4:** Most representative biomineralising cyanobacterial strains cited in the text. The table shows, for each cyanobacterial genus or species, the habitat, the mineral and the type of biomineralisation they have been reported to perform.

Genus/Species	Habitat type	Mineral type	Biomineralisation mode	References
*Synechocystis* sp. PCC 6803	Freshwater, lab model	CaCO_3_ (low)	BIEM mostly studied, not prominent	Han et al. (2013, 2017, 2022)
*Synechococcus calcipolaris*	Thermal springs	Intracellular CaCO_3_	BCIM	Liang et al. (2013); Benzerara et al. (2014)
*Gloeomargarita lithophora*	Alkaline environments	Intracellular CaCO_3_	BCIM	Benzerara et al. (2014)
*Microcystis* spp.	Freshwater (blooms)	Extracellular CaCO_3_	BIEM	Bruley et al. (2025)
*Synechococcus* spp.	Marine	CaCO_3_, SiO_2_	BIEM	Görgen et al. (2021)
*Nostoc*, *Oscillatoria*, *Phormidium*	Stromatolitic mats	CaCO_3_	BIEM	de Brito et al. (2023)
*Gloeothece*	Various	Unknown	Likely both	Jansson and Northen (2010)

Extracellular calcification is the most widespread form of biomineralisation in cyanobacteria, supported by the abundance of calcium in aquatic and terrestrial habitats. *Synechococcus* strains can precipitate CaCO_3_ under controlled calcium and carbon concentrations (Lee et al. 2006; Obst et al. 2009) (Table 4).

The pattern of carbonate deposition varies with cell morphology and colony structure. In *Gloeocapsa* sp., CaCO_3_ forms nanospheres near the cell surface (Bundeleva et al. 2014). Crystals may exhibit specific orientation, growing perpendicular to the cell surface, either inward or outward, often interacting with the surrounding EPS and biofilm matrix (Couradeau et al. 2013), suggesting that cell surface (EPS and S‐layer) properties act as a template for nucleation and mineral organisation (Figures 2 and 3b).

BIEM may provide physiological benefits: it protects from light damage, enhances nutrient uptake and helps regulate signalling pathways (Jansson and Northen 2010; McConnaughey and Whelan 1997). A key feature of BIEM in cyanobacteria is the ability to alter the immediate cell environment, particularly by raising the local environmental pH through photosynthesis, promoting favourable conditions for mineral precipitation. This process also helps regulate ionic balance, creates suitable microhabitats, shields cells from UV radiation, buffers pH fluctuation, and concentrates carbonates to enhance carbon fixation.

A relevant phenomenon of extracellular biomineralisation is represented by whitings (Figure 3). In this process, photosynthesis raises local pH of the surrounding environment, triggering the rapid precipitation of fine white CaCO_3_ microcrystals, typically as aragonite or calcite. These crystals remain suspended in water, producing a cloudy or milky appearance (Larson and Mylroie 2014; Sondi and Juračić 2010; Thompson et al. 1997). Unlike intracellular biomineralisation, whitings are an indirect byproduct of photosynthesis and do not seem to confer a direct metabolic benefit. Taken together, the selective advantage of extracellular biomineralisation likely results from multiple coexisting benefits that collectively enhance fitness.

### 3.3 Biologically Controlled Intracellular Mineralisation (BCIM): Topology, Diversity, and Functions

In BCIM, mineral formation is tightly regulated and typically occurs within specialised cellular regions or compartments. Unlike CEMI and BIEM, BCIM represents a distinct form of active and genetically encoded biomineralisation, directly coupled to specific metabolic states and often involving compartmentalisation within vesicles or other intracellular structures. The number of cyanobacterial species reported to form intracellular carbonates continues to increase (Blondeau et al. 2018; Cam et al. 2018; Segovia‐Campos et al. 2022), including model organisms such as *Thermosynechococcus elongatus* and *Synechocystis* spp. (Görgen et al. 2021). Particularly notable are *Gloeomargarita lithophora*, which forms intracellular carbonate inclusions under genetic control (Benzerara et al. 2014; Moreira et al. 2017), and the *Chroococcales* species *Microcystis aeruginosa* PCC 7806, which produces intracellular Amorphous Calcium‐rich Carbonates (iACC) in correlation with photosynthetic cycles and photoperiods, indicating strong dependence on metabolic state (Bruley et al. 2025) (Figure 3c).

Distinct underlying mechanisms emerge from the spatial localisation of intracellular mineral inclusions. *Gloeomargarita lithophora* shows randomly distributed inclusions, whereas *Synechococcus calcipolaris* forms inclusions restricted to the cell poles (Table 4). Such spatial patterns suggest links between biomineralisation, cell division (Benzerara et al. 2014; Li et al. 2016) and intracellular structures such as the cytoskeleton. Similarly, carboxysome and cell division proteins such as the tubulin‐like FtsZ may help guide the formation and localisation of these inclusions, as shown by their spatial proximity using electron microscopy (Li et al. 2016).

The composition and spatial arrangement of crystalline or amorphous intracellular minerals suggest a tightly regulated biochemical control. Several ecological and physiological hypotheses have been proposed. For example, inclusions may act as ballast for regulating cell dispersion in aquatic environments and/or they may result from photosynthetic activity as sinks for excess alkalinity, functioning as intracellular pH buffering systems (Couradeau et al. 2012).

Overall, BCIM likely reflects specific and often coexisting metabolic needs, which are as diverse as the biodiversity within this phylum. For instance, although CaCO_3_ minerals are the most commonly found, intracellular amorphous carbonates containing magnesium, strontium, or barium have also been reported (Couradeau et al. 2012), highlighting a variability in origin, composition, structure, and morphology across the phylum (Couradeau et al. 2012; Li et al. 2016). Cyanobacteria strains may produce differing numbers of inclusions per cell, which can be amorphous or crystalline (Blondeau et al. 2018; Cam et al. 2018; De Wever et al. 2019). Crystal morphology also varies significantly, from needle‐shaped to prismatic forms, with prismatic crystals generally larger (Figure 3c, Table 3).

Despite recent advances in identifying general functional features, the biological role of BCIM remains unclear. Nonetheless, its genetic regulation and association with specific physiological states strongly suggest functional relevance. Intracellular biominerals may participate in ion storage, intracellular pH buffering, or redox regulation. Given the metabolic investment and specificity required, BCIM likely represents a specialised adaptation shaped by evolutionary pressures, possibly linked to niche differentiation or enhanced resilience to environmental stress.

## 4 The Origin of Cyanobacterial Biomineralisation From Early Earth to Global Biogeochemical Cycles

Cyanobacteria are directly linked to the first organisms that marked the transition from a reducing to an oxidising atmosphere. This transformation represents a critical turning point in Earth's history and provides the foundation for understanding biomineralisation (Buick 2008; Riding 2006; Schopf 2006). Before this evolutionary milestone, biodiversity and biological specialisation were limited (Knoll 2015). Early life largely relied on passive interaction with the environment; therefore, mechanisms to obtain and exploit reservoirs of nutrients, such as crystalline or amorphous minerals of geological origin, could represent a selective advantage. Thus, the origins of biomineralisation must be traced to these early passive processes (Dupraz et al. 2004). These initial mechanisms modified the immediate surroundings of cells by altering local chemical conditions or forming solid, organised structures such as microbial mats. These mats created protective micro‐environments and enhanced habitability under conditions far more hostile than today (Bosak et al. 2013; Riding 2000).

Modern biomineralisation processes likely evolved from these early adaptive strategies, in which organisms initially enhanced their survival by passively exploiting available geological mineral reservoirs and later by actively modifying their surrounding environment.

Among microbial mats, the cyanobacterial ones are an example of such an evolutionary process and played a central role in global carbon fixation, enabling CO_2_ uptake and storage during early Earth conditions when inorganic carbon was abundant but not necessarily bioavailable due to limited enzymatic machineries and prevailing geochemical constraints (Falkowski et al. 2008; Raven et al. 2017). Throughout geological time, cyanobacteria have been drivers of the major biogeochemical cycles (Butterfield 2015). Their activity was fundamental not only in triggering the GOE (Lyons et al. 2014) but also in mobilisation and cycling of carbon on a planetary scale (Kump 2008) (Figure 1).

As a result of these evolutionary processes, cyanobacteria developed the capacity to promote and enhance mineral nucleation, giving rise to biomineralisation as a means of influencing nutrient availability, including carbon and optimising the habitability of their surrounding environment. This capacity is closely tied to the architecture of their outermost cell wall layers, which directly interface with the environment. These layers consist of repeating networks of modified sugars anchored to a para‐crystalline, proteinaceous S‐layer, providing an optimal topology for controlled mineral nucleation (Figure 2) (De Yoreo and Dove 2004; Westall et al. 2001). The regularity in space and charge distribution provided by the S‐layer likely contributes to the creation of an isotropic surface, a micro‐environment that buffers external fluctuations and supports sustained mineral growth, thus behaving as a scaffold. This implies that cyanobacteria modify their surroundings not only through metabolism by inducing, for example, CaCO_3_ precipitation by supersaturation (e.g., by EPS secretion or by photosynthesis) (Dittrich and Sibler 2005; Merz 1992), but also through molecular infrastructures that shape local chemical–physical conditions leading to optimised micro‐environments (Arp et al. 2001; De Yoreo and Dove 2004; Ercole et al. 2007; Westall et al. 2001). Minerals precipitated in this way were likely crucial in the formation of stromatolites, the layered mineral deposits within microbial mats that provide insights into Earth's early biosphere (Grotzinger and Knoll 1999; Landing and Johnson 2024).

## 5 Diverse Environmental Patterns Influence Biomineralisation Variety in Cyanobacteria

Biomineralisation is widespread among cyanobacteria, although not universally across all strains. It has been documented in multiple phylogenetic lineages, including well‐known genera such as *Synechocystis*, *Synechococcus* and *Microcystis*, as well as less‐studied genera like *Gloeothece and Chroococcidiopsis* (Table 4). These biomineralising strains originate from diverse environments such as marine, freshwater, thermal, and terrestrial habitats (Jansson and Northen 2010; Karlsson et al. 1983; Liang et al. 2013; Vaara 1982). Among them, the model cyanobacterium *Synechocystis* sp. PCC 6803 is widely used for biomineralisation studies (Kamennaya et al. 2012). Although it does not mineralise as prominently as other species, its genetic accessibility, well‐characterised physiology and experimental versatility have made it a preferred model, and the majority of biomineralisation studies in cyanobacteria have been conducted either on this strain specifically or within the *Synechocystis* genus (Han et al. 2013, 2022; Jiang et al. 2013; Zhao et al. 2023). Experiments on these organisms demonstrated the environmental influence on biomineralisation, revealing that it is highly sensitive to external conditions. Factors such as calcium, magnesium availability, light intensity and other physicochemical variables can influence both the mineral type and rate of precipitation (Han et al. 2017; Jiang et al. 2013; Zhao et al. 2023; Zhu et al. 2018).

When assessing biomineralisation in cyanobacteria, it is important to consider its distribution across environments given their differences in stability and fluctuations. Particularly informative is the frequent occurrence of biomineralising strains among extremophiles, suggesting that both the extent and nature of biomineralisation may be linked to strategies aimed at withstanding harsh or variable conditions (Benzerara et al. 2014; Ehrlich et al. 2021; Liang et al. 2013; Zhang 2023). Understanding how these ecophysiological properties, which coexist with biomineralisation, map onto phylogeny is also crucial for reconstructing how environmental pressures have shaped the evolutionary pathways of biomineralisation. While information in this area remains limited, several descriptors and general patterns can nevertheless be identified.

Inverting the perspective, biomineralisation in cyanobacteria involves species distributed across diverse environmental contexts ranging from stable aquatic ecosystems to highly fluctuating or extreme habitats. In particular, intracellular CaCO_3_, one of the most common inclusions, has been observed in phylogenetically diverse strains from marine, freshwater, thermal, and arid environments. These inclusions occur in crystalline or amorphous forms depending on the environment (Benzerara et al. 2014; Chrismas et al. 2015; Whitton and Potts 2012), and, even if with differences, their widespread presence suggests a fundamental physiological function rather than a mere stress‐induced phenomenon.

Environmental variability appears to play a crucial role in shaping biomineralisation processes, which in turn reflect broader physiological adaptations. For example, studies of cyanobacterial communities in the Atacama Desert show that these microorganisms interact with iron minerals of geological origin (such as magnetite and haematite) and can even influence crystal dimensions, eventually suggesting a mineral‐dependent adaptive mechanism driven by the environment that enhances their resilience in arid, mineral‐rich conditions (Huang et al. 2022). Likewise, several biomineralising cyanobacteria are extremophiles inhabiting hyper‐saline, thermal, or highly desiccated environments. *Chroococcidiopsis*, a genus thriving in deserts and polar regions, forms intracellular CaCO_3_ inclusions that may contribute to osmoregulation or enhance protection against desiccation, adaptations directly shaped by environmental pressures (Benzerara et al. 2022) (Table 4).

From a phylogenetic perspective, biomineralisation is shown to be distributed across multiple cyanobacterial lineages. A large‐scale screening of 68 strains revealed that intracellular CaCO_3_ biomineralisation is widespread throughout the phylum, suggesting an ancient and deeply rooted evolutionary origin shared through different environments (Benzerara et al. 2014). The identification of molecular markers such as the ccyA gene, encoding calcyanin, a protein associated with intracellular CaCO_3_ formation and thus involved in BCIM processes, further supports the presence of conserved genetic mechanisms shaped by the environment underlying this capacity (Benzerara et al. 2022).

Several well‐documented examples of crystal formation associated with cyanobacteria involve BIEM processes. These crystals often nucleate on the EPS within the mucilaginous sheaths of colonies, influencing water chemistry and contributing to sediment formation (Zhang 2023). Thus, it emerges a feedback mechanism by which the cell modifies its surrounding environment, and these environmental changes, in turn, not only induce biomineralisation but also affect its quality. As a specific example, in freshwater environments, *Microcystis* spp. induce CaCO_3_ precipitation during bloom events through photosynthesis‐driven increases in environmental pH, a phenomenon known as alkaline‐induced precipitation, which leads to carbonate supersaturation. Differently, in saltwater environments, *Synechococcus* spp. promote extracellular precipitation of both CaCO_3_ and silica, contributing to the biogeochemical cycling of carbon and silicon in oceanic ecosystems (de Brito et al. 2023; Tang et al. 2014). Perhaps the most striking example is stromatolite‐like forming cyanobacteria such as *Nostoc, Phormidium,* and *Oscillatoria*. Within microbial mats, these filamentous genera contribute to the layered deposition of CaCO_3_, a hallmark of stromatolite development and environmental shaping through metabolic activity. This form of extracellular biomineralisation represents one of the oldest biologically mediated mineralisations known, with modern analogues persisting today, notably in Shark Bay, Western Australia (Reid et al. 2003) (Table 4).

Although phylogenetic and environmental correlations remain incomplete, the available evidence suggests that environmental extremes and/or fluctuations act as selective pressures enhancing metabolic plasticity, which in turn drives the evolutionary diversification of biomineralisation in cyanobacteria.

## 6 The Interplay of S‐Layers, EPS, and Ion Homeostasis in Cyanobacterial Biomineralisation

Cyanobacteria are encased in a protective outer membrane whose outermost layers combine polysaccharides, which form the bulk of the EPS, with an underlying protein–lipid matrix building the S‐layer. The S‐layer and its associated EPS enable cyanobacteria to interact and influence their environment, with biomineralisation being the main evidence of it (Figure 2). Together, they protect cells from environmental stressors such as heat, desiccation and ultraviolet (UV) radiation while also facilitating the adsorption of metal ions and other dissolved constituents, creating localised micro‐environments that promote salt precipitation and mineral deposition. Although these structures are involved in mineral nucleation and in the formation of micro‐environments favourable for crystallisation of CaCO_3_, silica, or other minerals, their functional organisation and regulatory mechanisms remain partially understood, with multiple determinants still to be clarified (Benzerara et al. 2021; Schultze‐Lam et al. 1992; Schultze‐Lam and Beveridge 1994). In fact, several key biochemical pathways seem to be involved in modulating biomineralisation at the cell surface (de Brito et al. 2023). In particular, urease‐mediated alkalinisation and carbonic anhydrase activity locally increase pH and carbonate concentration, promoting CaCO_3_ nucleation. In this context, it is not only important the carbonates equilibrium but also the cation availability. Among the many ways through which calcium can be provided, the polyphosphate metabolism can mediate Ca^**2**+^ release while providing intracellular phosphorus storage. This process also relies on the Ca^**2**+^/H^+^ antiport systems that, by regulating periplasmic ion concentrations, contribute to supersaturation around the nucleation sites at the cell surface (Benzerara et al. 2022; Han et al. 2017; Jiang et al. 2013). In conclusion, the correlation between S‐layers, EPS and these metabolic pathways highlights the complex interlacements between cell structures, ion homeostasis, and extracellular mineral formation.

In fact, the EPS matrix acts as a scaffold that concentrates ions and guides mineral nucleation, such as CaCO_3_ or silica, while controlling the size and distribution of the resulting minerals. Consequently, the S‐layer, by acting as the anchoring base for the EPS and offering an isotropic surface with respect to structure and charges, provides an ideal platform for mineral nucleation. Its periodic electrostatic domains can bind calcium ions, creating a localised micro‐environment favouring CaCO_3_ saturation and precipitation. Imaging of S‐layer‐associated mineral deposits in *Synechocystis* supports the hypothesis that this layer actively directs mineral formation (Liang et al. 2013; Schultze‐Lam et al. 1992; Schultze‐Lam and Beveridge 1994), lowering the activation energy required for crystallisation. Consistently, potentiometric titration analysis has shown that changes in S‐layer charge density and in functional groups can influence the mineralisation rate and crystal morphology (Liang et al. 2013). This suggests that the surface composition may vary during growth phases or under stress, potentially disrupting biomineralisation and explaining its absence or rarity in certain phases.

The demonstrated metabolic dependence of biomineralisation naturally raises the question of whether cyanobacteria possess mechanisms to modulate mineral formation directly through metabolic regulation at the cell surface. Recent studies indicated that ion transporters and ion balance regulate mineral formation. In *Synechocystis* PCC 6803, inactivation of a Ca^2+^/H^+^ antiporters enhanced biomineralisation, likely through up‐regulation of CO_2_‐concentrating mechanisms and increased periplasmic alkalisation (Jiang et al. 2013). Similarly, Han et al. (2017) showed that low Mg^2+^/Ca^2+^ ratios induce both intracellular and extracellular biomineralisation in *Synechocystis*, highlighting the regulatory role of ionic conditions. Through photosynthesis, cyanobacteria produce oxygen, which can react with dissolved ions in the surrounding environment to promote mineral formation (Ferrini et al. 2021). Additionally, proteins such as calcyanin contribute to mineral precipitation (Benzerara et al. 2022). These observations, together with the ability of cyanobacteria to inhabit extreme environments such as alkaline lakes or hot springs while performing biomineralisation, suggest that, depending on the environmental conditions, they can actively mediate and modulate these processes at the cell–environment interface.

## 7 Conclusions on Cyanobacterial Biomineralisation in Global Ecological Resilience

Biomineralisation is a fundamental force in shaping Earth's environments and sustaining ecological balance. Given the broad implications of this process, it is difficult to clearly label biomineralisation as either positive or negative, especially when the underlying causes and contextual factors cannot be fully addressed. Therefore, a conservative interpretation is to see biomineralisation as a selective trait preserved by Darwinian evolution, meaning that if it has been conserved to the present day, it must provide a net positive benefit to the organism. Through biomineralisation, many organisms contribute to the formation of mineralised structures such as shells, skeletons, and microbial deposits (Lowenstam and Weiner 1989). These structures are essential for their survival and evolution, but also crucial, over long timescales, for the formation of coral reefs, limestone beds and stromatolites, all major components of the lithospheric carbon reservoir (Knoll 2015) (Figure 1). Through these formations, complex habitats have been developed, supporting diverse ecosystems and their interactions, shaping Earth's landscape, contributing to the long‐term carbon sequestration and helping regulate atmospheric composition (Dupraz and Visscher 2005). Ecologically, biomineralisation plays a central role in cycling elements such as carbon, nitrogen, sulphur, calcium, and silica. By incorporating carbon into mineral forms, biomineralising organisms help modulate global carbon levels and contribute to climate regulation (Arp et al. 2001). These processes buffer fluctuations in environmental chemistry by stabilising the pH, which in turn influences the availability of nutrients and trace metals, key factors for maintaining ecosystem stability, especially in marine environments.

In this context, cyanobacteria play a relevant role. These ancient microorganisms were among the first to engage in biomineralisation alongside oxygenic photosynthesis, significantly influencing early Earth environments (Schopf 2006). Their activity has shaped chemical and physical aspects of ecosystems across deep time. Although their role was crucial during the GOE, evidence suggests that the connection between cyanobacteria and biomineralisation began earlier, with the emergence of oxygenic photosynthesis in their ancestors. From their origin, these organisms likely contributed to biomineralisation, making it a continuous and fundamental ecological function (Buick 2008; Dupraz et al. 2004; Riding 2006; Schopf 2006). Over time, cyanobacterial biomineralisation became an essential mechanism for regulating, buffering, and cycling chemical elements in the biosphere. Through metabolic activity and mineral precipitation, they contribute to the sequestration of these elements, enabling their long‐term storage in sedimentary rocks, influencing local geochemical conditions and contributing to the long‐term regulation of Earth's atmospheric and oceanic chemistry (Figure 1). Biomineralisation is a biologically regulated process involving cellular structures, metabolic pathways, and environmental factors. By inducing CaCO_3_ precipitation, cyanobacteria alter their micro‐environment to promote mineral formation. The process is tightly linked to photosynthetically driven pH changes, ion transport and the architecture of the cyanobacterial cell envelope, which provides both physical templates and chemical sites for nucleation and crystal growth. Biomineralisation is also influenced by environmental stressors such as nutrient limitations and ion imbalances, suggesting roles in calcium regulation, carbon storage, or survival under unfavourable conditions. The flexibility of these responses highlights the evolutionary significance of biomineralisation in microbial adaptation to diverse and dynamic ecosystems. Taking all these factors into account, cyanobacteria help stabilise environmental conditions and support the resilience of ecosystems, promoting an ‘inertial ecology’ by resisting rapid changes and maintaining a functional continuity despite external disturbances.

Cyanobacterial biomineralisation also holds significant biotechnological potential, offering low‐energy, scalable and self‐sustaining strategies for carbon capture and long‐term sequestration (Falkenroth and Dann 2025). Applying the same rationale, biomineralisation in cyanobacteria also plays a primary role in bioremediation, enabling the precipitation and immobilisation of toxic metals, metalloids and even radionuclides in contaminated environments, effectively acting as natural buffering agents (Foster et al. 2020; Mehta et al. 2019). By converting hazardous substances into stable mineral phases, cyanobacteria reduce their mobility and bioavailability, contributing to detoxification and long‐term containment.

Despite advances in understanding biomineralisation, many questions remain regarding its genetic and molecular regulators, the ecological triggers that initiate it and the physiological role of mineral deposits. Future research should integrate knowledge on phylogeny, metabolism and cell‐envelope structures to understand cyanobacterial biomineralisation in specific environmental contexts. Distinguishing between different types of biomineralisation (BIEM, BCIM, CEMI) and their potential coexistence, while also linking them to the EPS/S‐layer system, metabolic traits and environmental parameters (e.g., calcium availability, carbonate chemistry, pH and supersaturation), will help clarify their functional roles and selective pressures (Benzerara et al. 2014; Han et al. 2017; Jiang et al. 2013; Obst et al. 2009; Paulo et al. 2018). Research should also explore how early passive mineralisation evolved into biologically controlled processes, connecting local adaptations to global ones such as stromatolite formation and carbon cycling (Falkowski et al. 2008; Grotzinger and Knoll 1999; Westall et al. 2001). The integration of BIEM, BCIM and CEMI with biodiversity and related ‘ecodiversity’ will reveal how cyanobacteria adapt their biomineralisation strategies to diverse habitats (Benzerara et al. 2014; Benzerara et al. 2022; Tang et al. 2014; Zhang 2023). Finally, mechanistic studies manipulating S‐layer, EPS, ionic conditions and metabolic activity will be essential to link specific cell structures and physiological processes to mineral formation and resilience in extreme or fluctuating environments (Ferrini et al. 2021; Liang et al. 2013; Schultze‐Lam et al. 1992).

Broadening our understanding of cyanobacterial biomineralisation will provide valuable insights into key questions related to early Earth processes, including the origins of life and the evolution of biogeochemical cycles over geological time. Consequently, continued interdisciplinary research will be essential for uncovering the full potential of these remarkable organisms, advancing our understanding of Earth's history (Allwood et al. 2006) and addressing contemporary environmental challenges.

## Author Contributions

**Federica Tiddia** and **Sandeesha Kodru:** writing – review and editing. **Dario Piano:** conceptualization, writing – original draft, writing – review and editing, visualization, funding acquisition. **Domenica Farci:** conceptualization, writing – original draft, writing – review and editing, funding acquisition, visualization, project administration, resources, supervision.

## Funding

This work was supported by Narodowe Centrum Nauki (PRO‐2022/47/D/NZ1/00126).

## Conflicts of Interest

The authors declare no conflicts of interest.

## Data Availability

Data sharing not applicable to this article as no datasets were generated or analysed during the current study.

## References

[emi470281-bib-0001] Allwood, A. C. , M. R. Walter , B. S. Kamber , C. P. Marshall , and I. W. Burch . 2006. “Stromatolite Reef From the Early Archaean Era of Australia.” Nature 441, no. 7094: 714–718. 10.1038/nature04764. 16760969

[emi470281-bib-0002] Arp, G. , A. Reimer , and J. Reitner . 2001. “Photosynthesis‐Induced Biofilm Calcification and Calcium Concentrations in Phanerozoic Oceans.” Science 292, no. 5522: 1701–1704. 10.1126/science.1057204. 11387471

[emi470281-bib-0003] Benzerara, K. , E. Duprat , T. Bitard‐Feildel , et al. 2022. “A New Gene Family Diagnostic for Intracellular Biomineralization of Amorphous ca Carbonates by Cyanobacteria.” Genome Biology and Evolution 14, no. 3: evac026. 10.1093/gbe/evac026. 35143662 PMC8890360

[emi470281-bib-0004] Benzerara, K. , J. Li , J. Miot , and F. Skouri‐Panet . 2021. “Calcium Carbonates Biomineralizations: Insights Into the Diversity and Evolution of Biomineralization Processes in Cyanobacteria.” Geobiology 19, no. 5: 535–552. 10.1111/gbi.12450.2021.

[emi470281-bib-0005] Benzerara, K. , J. Miot , G. Morin , G. Ona‐Nguema , F. Skouri‐Panet , and C. Férard . 2010. “Significance, Mechanisms and Environmental Implications of Microbial Biomineralization.” Comptes Rendus Geoscience 343, no. 2–3: 160–167. 10.1016/j.crte.2010.09.002.

[emi470281-bib-0006] Benzerara, K. , F. Skouri‐Panet , J. Li , et al. 2014. “Intracellular Ca‐carbonate Biomineralization Is Widespread in Cyanobacteria.” Proceedings of the National Academy of Sciences of The United States of America 111, no. 30: 10933–10938. 10.1073/pnas.1403510111. 25009182 PMC4121779

[emi470281-bib-0007] Blondeau, M. , M. Sachse , C. Boulogne , et al. 2018. “Amorphous Calcium Carbonate Granules Form Within an Intracellular Compartment in Calcifying Cyanobacteria.” Frontiers in Microbiology 6, no. 9: 1768. 10.3389/fmicb.2018.01768. PMC6087745 30127775

[emi470281-bib-0008] Bosak, T. , A. H. Knoll , and A. P. Petroff . 2013. “The Meaning of Stromatolites.” Annual Review of Earth and Planetary Sciences 41: 21–44. 10.1146/annurev-earth-042711-105327.

[emi470281-bib-0009] Bruley, A. , J. Gaëtan , M. Gugger , et al. 2025. “Diel Changes in the Expression of a Marker Gene and Candidate Genes for Intracellular Amorphous CaCO_3_ Biomineralization in Microcystis.” Peer Community Journal 5: e18. 10.24072/pcjournal.516.

[emi470281-bib-0010] Buick, R. 2008. “When Did Oxygenic Photosynthesis Evolve?” Philosophical Transactions of the Royal Society B 363: 2731–2743. 10.1098/rstb.2008.0041. PMC2606769 18468984

[emi470281-bib-0011] Bundeleva, I. A. , L. S. Shirokova , O. S. Pokrovsky , et al. 2014. “Experimental Modeling of Calcium Carbonate Precipitation by Cyanobacterium *Gloeocapsa* sp.” Chemical Geology 374–375: 44–60. 10.1016/j.chemgeo.2014.03.007.

[emi470281-bib-0012] Butterfield, J. 2015. “Early Evolution of the Eukaryota.” Palaeontology 58: 5–17. 10.1111/pala.12139.

[emi470281-bib-0013] Cam, N. , K. Benzerara , T. Georgelin , et al. 2018. “Cyanobacterial Formation of Intracellular ca‐Carbonates in Undersaturated Solutions.” Geobiology 16, no. 1: 49–61. 10.1111/gbi.12261. 29076282

[emi470281-bib-0014] Camacho, A. , X. A. Walter , A. Picazo , and J. Zopfi . 2017. “Photoferrotrophy: Remains of an Ancient Photosynthesis in Modern Environments.” Frontiers in Microbiology 8: 323. 10.3389/fmicb.2017.00323. 28377745 PMC5359306

[emi470281-bib-0015] Chrismas, N. A. , A. M. Anesio , and P. Sánchez‐Baracaldo . 2015. “Multiple Adaptations to Polar and Alpine Environments Within Cyanobacteria: A Phylogenomic and Bayesian Approach.” Frontiers in Microbiology 6: 1070. 10.3389/fmicb.2015.01070. 26528250 PMC4602134

[emi470281-bib-0016] Couradeau, E. , K. Benzerara , E. Gérard , et al. 2013. “Cyanobacterial Calcification in Modern Microbialites at the Submicrometer Scale.” Biogeosciences 10: 5255–5266. 10.5194/bg-10-5255-2013.

[emi470281-bib-0017] Couradeau, E. , K. Benzerara , D. Moreira , et al. 2011. “Prokaryotic and Eukaryotic Community Structure in Field and Cultured Microbialites From the Alkaline Lake Alchichica (Mexico).” PLoS One 6, no. 12: e28767. 10.1371/journal.pone.0028767. 22194908 PMC3237500

[emi470281-bib-0018] Couradeau, E. , K. Benzerara , D. Moreira , et al. 2012. “An Early‐Branching Microbialite Cyanobacterium Forms Intracellular Carbonates.” Science 336: 459–462. 10.1126/science.1216171. 22539718

[emi470281-bib-0019] de Brito, M. , I. Bundeleva , F. Marin , et al. 2023. “Properties of Exopolymeric Substances (EPSs) Produced During Cyanobacterial Growth: Potential Role in Whiting Events.” Biogeosciences 20: 3165–3183. 10.5194/bg-20-3165-2023.

[emi470281-bib-0020] De Wever, A. , K. Benzerara , M. Coutaud , et al. 2019. “Evidence of High ca Uptake by Cyanobacteria Forming Intracellular CaCO3 and Impact on Their Growth.” Geobiology 17, no. 6: 676–690. 10.1111/gbi.12358. 31347755

[emi470281-bib-0021] De Yoreo, J. J. , and P. M. Dove . 2004. “Materials Science.” Shaping Crystals With Biomolecules Science 306, no. 5700: 1301–1302. 10.1126/science.1100889. 15550649

[emi470281-bib-0022] Della, P. G. 2015. Carbonate Build‐Ups in Lacustrine, Hydrothermal and Fluvial Settings: Comparing Depositional Geometry, Fabric Types and Geochemical Signature, edited by D. W. J. Bosence , K. A. Gibbons , D. P. Le Heron , et al. Microbial Carbonates in Space and Time: Implications for Global Exploration and Production. 10.1144/SP418.4.

[emi470281-bib-0023] Dittrich, M. , and S. Sibler . 2005. “Cell Surface Groups of Two Picocyanobacteria Strains Studied by Zeta Potential Investigations, Potentiometric Titration, and Infrared Spectroscopy.” Journal of Colloid and Interface Science 286, no. 2: 487–495. 10.1016/j.jcis.2005.01.029. 15897062

[emi470281-bib-0024] Dupraz, C. , R. P. Reid , O. Braissant , A. W. Decho , R. S. Norman , and P. T. Visscher . 2009. “Processes of Carbonate Precipitation in Modern Microbial Mats.” Earth‐Science Reviews 96, no. 3–4: 141–162. 10.1016/j.earscirev.2008.10.005.

[emi470281-bib-0025] Dupraz, C. , and P. T. Visscher . 2005. “Microbial Lithification in Marine Stromatolites and Hypersaline Mats.” Trends in Microbiology 13, no. 9: 429–438. 10.1016/j.tim.2005.07.008. 16087339

[emi470281-bib-0026] Dupraz, C. , P. T. Visscher , L. K. Baumgartner , and R. P. Reid . 2004. “Microbe–Mineral Interactions: Early Carbonate Precipitation in a Hypersaline Lake (Eleuthera Island, Bahamas).” Sedimentology 51: 745–765. 10.1111/j.1365-3091.2004.00649.x.

[emi470281-bib-0027] Ehrlich, H. , E. Bailey , M. Wysokowski , and T. Jesionowski . 2021. “Forced Biomineralization: A Review.” Biomimetics (Basel) 6, no. 3: 46. 10.3390/biomimetics6030046. 34287234 PMC8293141

[emi470281-bib-0028] Ercole, C. , P. Cacchio , A. L. Botta , V. Centi , and A. Lepidi . 2007. “Bacterially Induced Mineralization of Calcium Carbonate: The Role of Exopolysaccharides and Capsular Polysaccharides.” Microscopy and Microanalysis 13, no. 1: 42–50. 10.1017/S1431927607070122. 17234036

[emi470281-bib-0029] Falkenroth, M. , and M. Dann . 2025. “Engineering Light‐Driven Biomineralization for a Sustainable Carbonate Economy.” Frontiers in Photobiology. 10.3389/fphbi.2025.1619812.

[emi470281-bib-0030] Falkowski, P. G. , T. Fenchel , and E. F. Delong . 2008. “The Microbial Engines That Drive Earth's Biogeochemical Cycles.” Science 320, no. 5879: 1034–1039. 10.1126/science.1153213. 18497287

[emi470281-bib-0031] Ferrini, T. , O. Grandjouan , O. Pourret , and R. E. Martinez . 2021. “Does Carbon Dioxide Storage by Cyanobacteria Induce Biomineralization in Presence of Basaltic Glass?” Geochemical Journal 55, no. 2: 51–58. 10.2343/geochemj.2.0617.

[emi470281-bib-0032] Foster, L. , K. Morris , A. Cleary , et al. 2020. “Biomineralization of Sr. by the Cyanobacterium *Pseudanabaena catenata* Under Alkaline Conditions.” Frontiers in Earth Science 8: 556244. 10.3389/feart.2020.556244.

[emi470281-bib-0033] Gérard, E. , B. Ménez , E. Couradeau , D. Moreira , K. Benzerara , and P. López‐García . 2013. “Specific Carbonate‐Microbe Interactions in the Modern Microbialites of Lake Alchichica (Mexico).” ISME Journal 7: 1997–2009. 10.1038/ismej.2013.81. 23804151 PMC3965311

[emi470281-bib-0034] Gilbert, B. , M. Abrecht , and C. W. Frazer . 2005. “The Organic–Mineral Interface in Biominerals.” In Reviews in Mineralogy & Geochemistry, edited by J. F. Banfield , vol. 59, 169–195. 10.2138/rmg.2005.59.7.

[emi470281-bib-0035] Görgen, S. , K. Benzerara , F. Skouri‐Panet , M. Gugger , F. Chauvat , and C. Cassier‐Chauvat . 2021. “The Diversity of Molecular Mechanisms of Carbonate Biomineralization by Bacteria.” Discover Materials 1: 2. 10.1007/s43939-020-00001-9.

[emi470281-bib-0036] Gross, M. 2015. “How Life Shaped Earth.” Current Biology 25, no. 19: R847–R850. 10.1016/j.cub.2015.09.011. 26726334

[emi470281-bib-0037] Grotzinger, J. P. , and A. H. Knoll . 1999. “Stromatolites in Precambrian Carbonates: Evolutionary Mileposts or Environmental Dipsticks?” Annual Review of Earth and Planetary Sciences 27: 313–358. 10.1146/annurev.earth.27.1.313. 11543060

[emi470281-bib-0038] Han, Z. , H. Yan , S. Zhou , et al. 2013. “Precipitation of Calcite Induced by *Synechocystis sp*. PCC6803.” World Journal of Microbiology and Biotechnology 29, no. 10: 1801–1811. 10.1007/s11274-013-1341-1. 23543209

[emi470281-bib-0039] Han, Z. , Y. Zhang , Y. Zhao , X. Gao , and M. E. Tucker . 2022. “Amorphous and Crystalline Carbonate Biomineralization in Cyanobacterial Biofilms Induced by *Synechocystis* sp. PCC6803 Cultured in CaCl_2_–MgCl_2_–SrCl_2_ Mediums.” Geomicrobiology Journal 39, no. 9: 767–780. 10.1080/01490451.2022.2074576.

[emi470281-bib-0040] Han, Z. , Y. Zhao , H. Yan , et al. 2017. “The Characterization of Intracellular and Extracellular Biomineralization Induced by *Synechocystis* sp. PCC6803 Cultured Under Low Mg/Ca Ratios Conditions.” Geomicrobiology Journal 34, no. 4: 362–373. 10.1080/01490451.2016.1197986.

[emi470281-bib-0041] Hazen, R. M. , D. Papineau , W. Bleeker , et al. 2008. “Review Paper. Mineral Evolution.” American Mineralogist 93, no. 11–12: 1693–1720. 10.2138/am.2008.2955.

[emi470281-bib-0042] Huang, W. , T. Wang , C. Perez‐Fernandez , J. DiRuggiero , and D. Kisailus . 2022. “Iron Acquisition and Mineral Transformation by Cyanobacteria Living in Extreme Environments.” Materials Today Bio 17: 100493. 10.1016/j.mtbio.2022.100493. PMC9682352 36438421

[emi470281-bib-0043] Jansson, C. , and T. Northen . 2010. “Calcifying Cyanobacteria—The Potential of Biomineralization for Carbon Capture and Storage.” Current Opinion in Biotechnology 21, no. 3: 365–371. 10.1016/j.copbio.2010.03.017. 20456936

[emi470281-bib-0044] Jehlička, J. , A. Oren , P. Vítek , and J. Wierzchos . 2024. “Microbial Colonization of Gypsum: From the Fossil Record to the Present Day.” Frontiers in Microbiology 15: 1397437. 10.3389/fmicb.2024.1397437. 39228380 PMC11368868

[emi470281-bib-0045] Jiang, H.‐B. , H.‐M. Cheng , K.‐S. Gao , and B.‐S. Qiu . 2013. “Inactivation of Ca^2+^/H^+^ Exchanger in *Synechocystis* sp. Strain PCC 6803 Promotes Cyanobacterial Calcification by Upregulating CO_2_‐Concentrating Mechanisms.” Applied and Environmental Microbiology 79: 4048–4055. 10.1128/AEM.00681-13. 23624472 PMC3697565

[emi470281-bib-0046] Kamennaya, N. A. , C. M. Ajo‐Franklin , T. Northen , and C. Jansson . 2012. “Cyanobacteria as Biocatalysts for Carbonate Mineralization.” Minerals 2: 338–364. 10.3390/min2040338.

[emi470281-bib-0047] Kanellopoulos, C. , V. Lamprinou , A. Politi , et al. 2022. “Microbial Mat Stratification in Travertine Depositions of Greek Hot Springs and Biomineralization Processes.” Minerals 12, no. 11: 1408. 10.3390/min12111408.

[emi470281-bib-0048] Karlsson, B. , T. Vaara , K. Lounatmaa , and H. Gyllenber . 1983. “Three‐Dimensional Structure of the Regularly Constructed Surface Layer From *Synechocystis* sp. Strain CLII.” Journal of Bacteriology 156, no. 3: 1338–1343. 10.1128/jb.156.3.1338-1343.1983. 6417112 PMC217985

[emi470281-bib-0049] Knoll, A. H. 2015. Life on a Young Planet: The First Three Billion Years of Evolution on Earth—. Updated ed. Princeton University Press.

[emi470281-bib-0050] Konhauser, K. O. 2007. Introduction to Geomicrobiology. (2nd ed.). ed. Blackwell Publishing.

[emi470281-bib-0051] Kump, L. R. 2008. “The Rise of Atmospheric Oxygen.” Nature 451, no. 7176: 277–278. 10.1038/nature06587. 18202642

[emi470281-bib-0052] Landing, E. , and M. E. Johnson . 2024. “Stromatolites and Their “Kin” as Living Microbialites in Contemporary Settings Linked to a Long Fossil Record.” Journal of Marine Science and Engineering 12, no. 12: 2127. 10.3390/jmse12122127.

[emi470281-bib-0053] Larson, E. B. , and J. E. Mylroie . 2014. “A Review of Whiting Formation in The Bahamas and New Models.” Carbonates and Evaporites 29: 337–347. 10.1007/s13146-014-0212-7.

[emi470281-bib-0054] Lee, B. D. , W. A. Apel , and M. R. Walton . 2006. “Calcium Carbonate Formation by *Synechococcus* sp. Strain PCC 8806 and *Synechococcus* sp. Strain PCC 8807.” Bioresource Technology 97, no. 18: 2427–2434. 10.1016/j.biortech.2005.09.028. 16289626

[emi470281-bib-0055] Li, J. , I. Margaret‐Oliver , N. Cam , et al. 2016. “Biomineralization Patterns of Intracellular Carbonatogenesis in Cyanobacteria: Molecular Hypotheses.” Minerals 6, no. 1: 10. 10.3390/min6010010.

[emi470281-bib-0056] Liang, A. , C. Paulo , Y. Zhu , and M. Dittrich . 2013. “CaCO_3_ Biomineralization on Cyanobacterial Surfaces: Insights From Experiments With Three *Synechococcus* Strains.” Colloids and Surfaces. B, Biointerfaces 111: 600–608. 10.1016/j.colsurfb.2013.07.012. 23899673

[emi470281-bib-0057] Lowenstam, H. A. , and S. Weiner . 1989. On Biomineralization. Oxford University Press.

[emi470281-bib-0058] Ludwig, W. , and K. H. Schleifer . 1994. “Bacterial Phylogeny Based on 16S and 23S rRNA Sequence Analysis.” FEMS Microbiology Reviews 15, no. 2–3: 155–173. 10.1111/j.1574-6976.1994.tb00132.x. 7524576

[emi470281-bib-0059] Lyons, T. W. , C. T. Reinhard , and N. J. Planavsky . 2014. “The Rise of Oxygen in Earth's Early Ocean and Atmosphere.” Nature 506, no. 7488: 307–315. 10.1038/nature13068. 24553238

[emi470281-bib-0060] Magnabosco, C. , F. Husain , M. M. Paoletti , et al. 2024. “Toward a Natural History of Microbial Life.” Annual Review 52: 85–108. 10.1146/annurev-earth-031621-070542.

[emi470281-bib-0061] McConnaughey, T. A. , and J. F. Whelan . 1997. “Calcification Generates Protons for Nutrient and Bicarbonate Uptake.” Earth‐Science Reviews 42, no. 1–2: 95–117. 10.1016/S0012-8252(96)00036-0.

[emi470281-bib-0062] McCutcheon, J. , I. M. Power , A. L. Harrison , G. M. Dipple , and G. Southam . 2014. “A Greenhouse Scale Photosynthetic Microbial Bioreactor for Carbon Sequestration in Magnesium Carbonate Minerals.” Environmental Science & Technology 48, no. 16: 9142–9151. 10.1021/es500344s. 25072950

[emi470281-bib-0063] Mehdizadeh Allaf, M. , and H. Peerhossaini . 2022. “Cyanobacteria: Model Microorganisms and Beyond.” Microorganisms 10, no. 4: 696. 10.3390/microorganisms10040696. 35456747 PMC9025173

[emi470281-bib-0064] Mehta, N. , K. Benzerara , B. D. Kocar , and V. Chapon . 2019. “Sequestration of Radionuclides Radium‐226 and Strontium‐90 by Cyanobacteria Forming Intracellular Calcium Carbonates.” Environmental Science & Technology 53, no. 21: 12639–12647. 10.1021/acs.est.9b03982. 31584265

[emi470281-bib-0065] Merz, M. U. E. 1992. “The Biology of Carbonate Precipitation by Cyanobacteria.” Facies 26: 81–101. 10.1007/BF02539795.

[emi470281-bib-0066] Moreira, D. , R. Tavera , K. Benzerara , et al. 2017. “Description of *Gloeomargarita Lithophora* Gen. Nov., sp. Nov., a Thylakoid‐Bearing, Basal‐Branching Cyanobacterium With Intracellular Carbonates, and Proposal for *Gloeomargaritales* Ord.” International Journal of Systematic and Evolutionary Microbiology 67, no. 3: 653–658. 10.1099/ijsem.0.001679. 27902306 PMC5669459

[emi470281-bib-0067] Obst, M. , B. Wehrli , and M. Dittrich . 2009. “CaCO_3_ Nucleation by Cyanobacteria: Laboratory Evidence for a.Passive, Surface‐Induced Mechanism.” Geobiology 7, no. 3: 324–347. 10.1111/j.1472-4669.2009.00200.x. 19476505

[emi470281-bib-0068] Olejarz, J. , Y. Iwasa , A. H. Knoll , and M. A. Nowak . 2021. “The Great Oxygenation Event as a Consequence of Ecological Dynamics Modulated by Planetary Change.” Nature Communications 12, no. 1: 3985. 10.1038/s41467-021-23,286-7. PMC8238953 34183660

[emi470281-bib-0069] Paulo, C. , J. P. L. Kenney , P. Persson , and M. Dittrich . 2018. “Effects of Phosphorus in Growth Media on Biomineralization and Cell Surface Properties of Marine Cyanobacteria *Synechococcus* .” Geosciences 8: 471. 10.3390/geosciences8120471.

[emi470281-bib-0070] Popall, R. M. , H. Bolhuis , G. Muyzer , and M. Sánchez‐Román . 2020. “Stromatolites as Biosignatures of Atmospheric Oxygenation: Carbonate Biomineralization and UV‐C Resilience in a *Geitlerinema* sp. ‐ Dominated Culture.” Frontiers in Microbiology 11: 948. 10.3389/fmicb.2020.00948. 32508777 PMC7248245

[emi470281-bib-0071] Ragon, M. , K. Benzerara , D. Moreira , R. Tavera , and P. López‐García . 2014. “16S rDNA‐Based Analysis Reveals Cosmopolitan Occurrence but Limited Diversity of Two Cyanobacterial Lineages With Contrasted Patterns of Intracellular Carbonate Mineralization.” Frontiers in Microbiology 5: 331. 10.3389/fmicb.2014.00331. 25071744 PMC4085569

[emi470281-bib-0072] Raven, J. A. , J. Beardall , and P. Sánchez‐Baracaldo . 2017. “The Possible Evolution and Future of CO_2_‐Concentrating Mechanisms.” Journal of Experimental Botany 68, no. 14: 3701–3716. 10.1093/jxb/erx110. 28505361

[emi470281-bib-0073] Reid, P. R. , J. N. P. James , I. G. Macintyre , C. P. Dupraz , and R. V. Burne . 2003. “Shark Bay Stromatolites: Microfabrics and Reinterpretation of Origins.” Facies 49: 299–324. 10.1007/s10347-003-0036-8.

[emi470281-bib-0074] Riding, R. 2000. “Microbial Carbonates: The Geological Record of Calcified Bacterial–Algal Mats and Biofilms.” Sedimentology 47: 179–214. 10.1046/j.1365-3091.2000.00003.x.

[emi470281-bib-0075] Riding, R. 2006. “Cyanobacterial Calcification, Carbon Dioxide Concentrating Mechanisms, and Proterozoic–Cambrian Changes in Atmospheric Composition.” Geobiology 4, no. 4: 299–316. 10.1111/j.1472-4669.2006.00087.x.

[emi470281-bib-0076] Schopf, J. W. 2006. “Fossil Evidence of Archaean Life.” Philosophical Transactions of the Royal Society B 361, no. 1470: 869–885. 10.1098/rstb.2006.1834. PMC1578735 16754604

[emi470281-bib-0077] Schultze‐Lam, S. , and T. J. Beveridge . 1994. “Physicochemical Characteristics of the Mineral‐Forming S‐Layer From the Cyanobacterium *Synechococcus* Strain GL24.” Canadian Journal of Microbiology 40, no. 3: 216–223. 10.1139/m94-035.s.

[emi470281-bib-0078] Schultze‐Lam, S. , G. Harauz , and T. J. Beveridge . 1992. “Participation of a Cyanobacterial S‐Layer in Fine‐Grain Mineral Formation.” Journal of Bacteriology 174, no. 24: 7971–7981. 10.1128/jb.174.24.7971-7981.1992. 1459945 PMC207533

[emi470281-bib-0079] Segovia‐Campos, I. , A. Martignier , M. Filella , J. M. Jaquet , and D. Ariztegui . 2022. “Micropearls and Other Intracellular Inclusions of Amorphous Calcium Carbonate: An Unsuspected Biomineralization Capacity Shared by Diverse Microorganisms.” Environmental Microbiology 24, no. 2: 537–550. 10.1111/1462-2920.15498. 33817930 PMC9292747

[emi470281-bib-0080] Sondi, I. , and M. Juračić . 2010. “Whiting Events and the Formation of Aragonite in Mediterranean Karstic Marine Lakes: New Evidence on Its Biologically Induced Inorganic Origin.” Sedimentology 57: 85–95. 10.1111/j.1365-3091.2009.01090.x.

[emi470281-bib-0081] Spitzer, S. , N. Brinkmann , A. Reimer , et al. 2015. “Effect of Variable pCO_2_ on Ca^2+^ Removal and Potential Calcification of Cyanobacterial Biofilms—An Experimental Microsensor Study.” Geomicrobiology Journal 32, no. 3–4: 304–315. 10.1080/01490451.2014.885617.

[emi470281-bib-0082] Svirčev, Z. , T. Dulić , I. Obreht , et al. 2019. “Cyanobacteria and Loess—An Underestimated Interaction.” Plant and Soil 439: 293–308. 10.1007/s11104-019-04048-3.

[emi470281-bib-0083] Swanner, E. , A. Mloszewska , O. Cirpka , R. Schoenberg , K. O. Konhauser , and A. Kappler . 2015. “Modulation of Oxygen Production in Archaean Oceans by Episodes of Fe(II) Toxicity.” Nature Geoscience 8: 126–130. 10.1038/ngeo2327.

[emi470281-bib-0084] Tang, T. , K. Kisslinger , and C. Lee . 2014. “Silicate Deposition During Decomposition of Cyanobacteria May Promote Export of Picophytoplankton to the Deep Ocean.” Nature Communications 5: 4143. 10.1038/ncomms5143. 24920300

[emi470281-bib-0085] Thompson, J. B. , S. Schultze‐Lam , T. J. Beveridge , and D. J. Des Marais . 1997. “Whiting Events: Biogenic Origin due to the Photosynthetic Activity of Cyanobacterial Picoplankton.” Limnology and Oceanography 42, no. 1: 133–141. 10.4319/lo.1997.42.1.0133. 11541205

[emi470281-bib-0086] Tice, M. M. , and D. R. Lowe . 2004. “Photosynthetic Microbial Mats in the 3416‐Myr‐Old Ocean.” Nature 431, no. 7008: 549–552. 10.1038/nature02888. 15457255

[emi470281-bib-0087] Vaara, T. 1982. “The Outermost Surface Structures in Chroococcacean Cyanobacteria.” Canadian Journal of Microbiology 28, no. 8: 929–941. 10.1139/m82-140.

[emi470281-bib-0088] Westall, F. , M. J. De Witb , J. Dann , S. Van Der Gaast , C. E. J. De Ronded , and D. Gerneke . 2001. “Early Archean Fossil Bacteria and Biofilms in Hydrothermally‐Influenced Sediments From the Barberton Greenstone Belt.” South Africa Precambrian Research 106: 93–116. 10.1016/S0301-9268(00)00127-3.

[emi470281-bib-0089] Whitton, B. A. , and M. Potts . 2012. “Introduction to the Cyanobacteria.” In Ecology of Cyanobacteria II: Their Diversity in Space and Time, edited by B. A. Whitton , 1–13. Springer ISBN: 9789400738546.

[emi470281-bib-0090] Woese, C. R. 1987. “Bacterial Evolution.” Microbiological Reviews 51, no. 2: 221–271. 10.1128/mr.51.2.221-271.1987. 2439888 PMC373105

[emi470281-bib-0091] Zhang, J. Z. 2023. “Cyanobacteria Blooms Induced Precipitation of Calcium Carbonate and Dissolution of Silica in a Subtropical Lagoon, Florida Bay, USA.” Scientific Reports 13, no. 1: 4071. 10.1038/s41598-023-30,905-4. 36906722 PMC10008547

[emi470281-bib-0092] Zhang, X. , M. Dai , M. Wang , and Y. Qi . 2019. “Calcified Coccoid From Cambrian Miaolingian: Revealing the Potential Cellular Structure of Epiphyton.” PLoS One 14, no. 3: e0213695. 10.1371/journal.pone.0213695. 30870473 PMC6417771

[emi470281-bib-0093] Zhao, H. , Y. Han , M. Liang , et al. 2023. “Effect of Magnesium and Ferric Ions on the Biomineralization of Calcium Carbonate Induced by *Synechocystis* sp.” PCC 6803. Minerals. 13(12):1486. 10.3390/min13121486.

[emi470281-bib-0094] Zhu, T. , Y. Lin , X. Lu , and M. Dittrich . 2018. “Assessment of Cyanobacterial Species for Carbonate Precipitation on Mortar Surface Under Different Conditions.” Ecological Engineering 120: 154–163. 10.1016/j.ecoleng.2018.05.038.

